# The Homologous Components of Flagellar Type III Protein Apparatus Have Acquired a Novel Function to Control Twitching Motility in a Non-Flagellated Biocontrol Bacterium

**DOI:** 10.3390/biom10050733

**Published:** 2020-05-07

**Authors:** Alex M. Fulano, Danyu Shen, Miki Kinoshita, Shan-Ho Chou, Guoliang Qian

**Affiliations:** 1 College of Plant Protection (Laboratory of Plant Immunity; Key Laboratory of Integrated Management of Crop Diseases and Pests), Nanjing Agricultural University, Nanjing 210095, China; fluxali.alex@gmail.com (A.M.F.); shendanyu@njau.edu.cn (D.S.); 2 Graduate School of Frontier Biosciences, Osaka University, 1-3 Yamadaoka, Suita, Osaka 565-0871, Japan; miki@fbs.osaka-u.ac.jp; 3 Institute of Biochemistry, and NCHU Agricultural Biotechnology Center, National Chung Hsing University, Taichung 402, Taiwan; shchou@nchu.edu.tw

**Keywords:** flagellar type III apparatus, type IV pilus, non-flagellated bacteria, *Lysobacter*, twitching motility

## Abstract

The bacterial flagellum is one of the best-studied surface-attached appendages in bacteria. Flagellar assembly in vivo is promoted by its own protein export apparatus, a type III secretion system (T3SS) in pathogenic bacteria. *Lysobacter enzymogenes* OH11 is a non-flagellated soil bacterium that utilizes type IV pilus (T4P)-driven twitching motility to prey upon nearby fungi for food. Interestingly, the strain OH11 encodes components homologous to the flagellar type III protein apparatus (FT3SS) on its genome, but it remains unknown whether this FT3SS-like system is functional. Here, we report that, despite the absence of flagella, the FT3SS homologous genes are responsible not only for the export of the heterologous flagellin in strain OH11 but also for twitching motility. Blocking the FT3SS-like system by in-frame deletion mutations in either *flhB* or *fliI* abolished the secretion of heterologous flagellin molecules into the culture medium, indicating that the FT3SS is functional in strain OH11. A deletion of *flhA*, *flhB*, *fliI*, or *fliR* inhibited T4P-driven twitching motility, whereas neither that of *fliP* nor *fliQ* did, suggesting that FlhA, FlhB, FliI, and FliR may obtain a novel function to modulate the twitching motility. The flagellar FliI ATPase was required for the secretion of the major pilus subunit, PilA, suggesting that FliI would have evolved to act as a PilB-like pilus ATPase. These observations lead to a plausible hypothesis that the non-flagellated *L. enzymogenes* OH11 could preserve FT3SS-like genes for acquiring a distinct function to regulate twitching motility associated with its predatory behavior.

## 1. Introduction

*Lysobacter enzymogenes* is a Gram-negative, environmentally ubiquitous bacterium [1]. It was shown that this bacterium produces numerous anti-infectious metabolites and extracellular lytic enzymes [1,2,3,4]. A distinct feature of *L. enzymogenes* is the evolutionary loss of a surface-attached flagellum, due to the lack of multiple flagellar biogenesis genes such as the *fliC* gene encoding the flagellin subunit [5]. This non-flagellated bacterium exhibits a twitching behavior in natural niches that is powered by type IV pilus (T4P) [6]. As a powerful agent against crop fungal pathogens, *L. enzymogenes* deploys the T4P-driven twitching motility to move towards ecologically relevant, filamentous fungi to prey on them as foods [2,7]. In the model strain OH11, we previously discovered that numerous pilus structural component proteins, including the major pilus subunit, PilA and the motor proteins PilB, and the outer membrane secretin PilQ, are required for the biogenesis of T4P and the function of twitching motility [7].

Flagellated bacteria usually use the flagellum consisting of a filament (helical propeller), a hook (universal joint), and a basal body (rotary motor), to migrate towards more suitable conditions and to escape from undesirable environments for ecological adaption and survival [8,9,10,11,12]. Flagellar assembly is a complicated process involving many flagellar building blocks exported beyond the cellular membranes. This assembly process is dependent upon the flagellar type III protein export apparatus (FT3SS) [13]. The FliI ATPase energizes the unfolding of substrates and disassembly of substrate/chaperone complexes to load them onto the export gate [13]. The export itself mainly works in a proton-motive force (PMF)-driven manner [14]. However, protein export is also possible in the absence of FliI, although less efficient [15]. Five highly conserved inner membrane proteins (FlhA, FlhB, FliP, FliQ, and FliR) form the transmembrane export gate complex that collaborates with the cytoplasmic ATPase ring complex (formed by FliH, FliI, and FliJ) for energy transduction to transport flagellar proteins from the cytoplasm to the distal end of the growing flagellar structure [10,14,15,16,17,18,19]. Two of the transmembrane export gate complex components, FlhA and FlhB, are involved in substrate (hook-type and filament-type proteins) specificity switching [20,21,22,23]. In general, FT3SS components are commonly distributed among flagellated bacteria and are mainly responsible for flagellar protein export, thereby playing an indispensable role inflagellar-driven motility [22]. Recently, a divergent function of FT3SS has been observed in *Bacillus subtilis*, a flagellated, Gram-positive bacterium. In this bacterium, the FT3SS gene products participate in forming flagella-independent nanotubes that are involved in cell-cell exchange of proteins or plasmids [24].

The non-flagellated strain OH11 encodes flagellar FT3SS-like genes on its genome. To clarify whether FT3SS is functional in the non-flagellated strain OH11, we disrupted each homologous gene and analyzed whether deletion mutant strains can transport heterologous FliC molecules (also known as flagellin) derived from *Xanthomonas oryzae*, a flagellated, closely related species of *L. enzymogenes*. We show that mutations in two selected FT3SS-like genes in the wild-type OH11 blocked the secretion of the heterologous flagellin molecules. These findings suggest that despite the lack of a flagellum, strain OH11 still seems to retain anFT3SS-like system that could mediate the secretion of the heterologous flagellar proteins with functions similar to those of canonical flagellar FT3SS. We further show that four FT3SS-like proteins (FlhA, FlhB, FliI, and FilR) in strain OH11 are required for T4P-driven twitching motility. In particular, the flagellar FliI homolog in strain OH11 acts as a PilB-like ATPase to facilitate the secretion of PilA. Our results demonstrate that several FT3SS-like genes that are potentially vestigial in the non-flagellated *L. enzymogenes* appear to be required for T4P-driven twitching motility, highlighting the functional divergence of the FT3SS genes in flagellated and non-flagellated bacteria. Our findings also build on recent work from others that FT3SS play roles in bacterial physiology beyond flagella production.

## 2. Materials and Methods

### 2.1. Bacterial Strains, Plasmids and Culture Conditions

The bacterial strains and plasmids used in this study are listed in Table S1. *Escherichia coli* strain DH5α was used for vector construction and was grown in Luria Bertani (LB) broth at 37 °C. Unless otherwise stated, the wild-type *L. enzymogenes* OH11 and its derivatives were grown in LB medium at 28 °C. When required, the medium was amended with gentamicin (Gm) and kanamycin (Km) atfinal concentrations of 150 μg/mL and 100 μg/mL, respectively.

### 2.2. Genetic Manipulation

The in-frame deletion mutants of the FT3SS genes of strain OH11 have been generated and stored in the laboratory. Recombinant plasmids for complementation were constructed according to our earlier reports [25,26]. In summary, the *flhA*, *flhB*, *fliI*, and *fliR* DNA fragments, each containing full-length gene and its predicated promoter region, were amplified by PCR with different conjugated primer pairs (Table S2). Promoter prediction analysis was conducted with prediction programs [27]. Each amplified DNA fragment was cloned into the broad-host vector pBBR1-MCS5. The resulting recombinant plasmids, pBBR-*flhA*, pBBR-*flhB*, pBBR-*fliI*, and pBBR-*fliR* were individually transformed into competent cells of Δ*flhA*, Δ*flhB*, Δ*fliI*, and Δ*fliR* by electroporation, respectively. The resulting clones were screened by colony PCR and further validated by sequencing. The same approach was used in constructing pBBR-*pilA*, which was introduced to various strains including wild-type *L. enzymogenes* OH11 as listed in Table S1.

For cross complementation, each predicted FT3SS-like gene from strain OH11 was amplified by PCR with the primers listed in Table S2 and cloned into the vector of pTrc99A (Table S1). The resultant recombinant plasmids were individually introduced into the *Salmonella* mutants lacking the respective flagellar counterpart genes (Table S1). The flagellum-driven swimming motility assay was performed as described in [14]. For the expression of heterologous flagellar protein in strain OH11, the broad-host vector, pBBR1-MCS5 was cloned with the *fliC_Xoo_* gene from *Xanthomonas oryzae* pv. *oryzae* PX099A (GenBank accession no. NC_010717.2) fused with a Flag tag (Table S1). The resulting plasmid was transformed into respective competent cells of in-frame deletion mutants of *L. enzymogenes* OH11 by electroporation. The resulting clones were screened by colony PCR and further validated by sequencing.

### 2.3. Twitching Motility Assay

Twitching motility assays were carried out as described previously [6,7]. In brief, *L. enzymogenes* OH11 and its derivatives were grown in 1/20 tryptic soy broth (TSB) agar to a cell density of OD_600_ 1. We aseptically moistened a piece of blotting paper by flushing 750 μL of sterile de-ionized water on the left and right side of a glass slide. Then 20 μL of 5% TSA containing 1.8% agar was evenly spread onto a sterilized microscope slide placed on a humid blotter. The edge of a sterilized cover-slip was dropped into 1000 μL bacterial cell suspension in another glass Petri dish and then gently pressed onto the surface of the medium to create a thin inoculation line. After 24 h of incubation at 28 °C, the margin of the bacterial culture on the glass slide was observed under a microscope at ×640 magnification with images captured using an Olympus DP72 Camera (Olympus, Center Valley, PA, USA). The presence of individual mobile cells or small clusters of cells growing outwardly from the main colony wasan indication of twitching motility. Three independent experiments with each involving three replicates were carried out.

### 2.4. Immunoblotting

The wild-type OH11 and its derivative strains were grown to an OD_600_ 1.5. For detecting secretion of PilA-Flag, a protocol from a recent study [28] was adopted with slight modifications. The culture samples, each measuring 40 mL, were centrifuged for 30 min at a speed of 6000 rpm at 4 °C to separate cells and culture supernatants. Supernatant fractions were then passed through 0.22-mm filters, with 10% *v*/*v* trichloroacetic acid added to the filtered supernatants, which were kept at 4 °C overnight to precipitate secreted proteins. The resultant supernatant precipitates were pelleted by centrifugation at 12,000 rpm for 20 min at 4 °C and subsequently washed in ice-cold acetone three times (centrifugation at 12,000 rpm for 15 min at 4 °C). The harvested supernatant precipitates were re-suspended in 1 mL sterile distilled water before subjecting them to centrifugation at 12,000 rpm for 15 min at 4 °C. For all cellular proteins in this study, we pelleted bacterial cells by centrifugation. The pellets were suspended in 2× SDS buffer to a final volume of 60 μL before addition of SDS-PAGE loading buffer. Both supernatant precipitates and cellular fractions were subjected to sodium dodecyl sulfate-polyacrylamide gel electrophoresis (SDS-PAGE) preceding Western blotting assay.

The proteins were transferred onto polyvinylidene difluoride (PVDF) membrane using a semi-dry blot machine (Bio-RAD, Hercules, CA, USA). The membrane was blocked in 5% (*w*/*v*) skim milk in 1× TBS for 1 h at room temperature or overnight at 4 °C with gentle constant rocking. This was then incubated with monoclonal antibody specific for the Flag tag (Anti-DYKDDDDK-Tag Mouse mAb, Abmart) at 1:10,000 dilution at room temperature for 1 h. Secondary HRP-conjugated goat anti-mouse antibody (Abmart) at the same dilution factor as the above primary antibody was applied after washing the membranes in TBST buffer (50 mM Tris, 150 mM NaCl, 0.05% (*v*/*v*) Tween 20, pH 7.4) three times at an interval of 10 min. For detecting expression of FT3SS components, we used monoclonal antibodies at 1:20,000 dilution. The secondary antibody used for the above primary monoclonal antibodies was PRP-Goat Anti-Rabbit IgG (H + C) from Jackson Immuno Research (West Grove, PA, USA). Three independent experiments with each involving three replicates were carried out.

## 3. Results

### 3.1. The Non-Flagellated L. enzymogenes OH11 Encodes FT3SS-Like Genes

**Figure 1:**
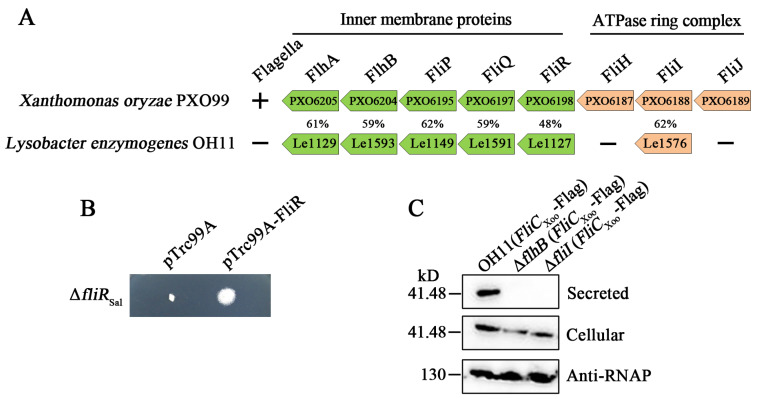
The non-flagellated *Lysobacter enzymognes* OH11 likely carried a functional flagellar type III apparatus (FT3SS) that was able to mediate the export of heterologous flagellar protein. (**A**) Presence of flagellar type III protein apparatus (FT3SS) homologs in the non-flagellated *Lysobacter enzymogenes* OH11. The FT3SS genes characterized in the flagellated *Xanthomonas oryze* PXO99A, a closely related species of *L. enzymogenes* that was used as reference. The sequence similarity between each pair of proteins was provided accordingly. + and −, flagella present and absent, respectively (**B**) The FliR homolog of strain OH11 partially restored the swimming motility of the *Salmonella fliR*_Sal_ null mutant. The photograph was taken after incubation of the transformed strain at 30 °C for 24 h. (**C**) Secretion of the heterologous FliC_Xoo_was dependent on the presence of FT3SS genes in strain OH11. The coding gene of FliC_Xoo_ from *X. oryzae* PXO99A was fused with a Flag-tag and introduced into the wild-type OH11 and two selected FT3SS-defective mutants (Δ*fllhB* and Δ*fliI*). The signal of the FliC_Xoo_-Flag fusion protein was detected in the culture supernatant (defined as “Secreted”) of wild-type OH11 but not the test mutant strains, while the intracellular presence (defined as “Cellular”) of FliC_Xoo_-Flag was observed in both wild type and two FT3SS-defective mutants.

The plant pathogenic *Xanthomonas oryzae* is a flagellated, taxonomically close bacterium with *L. enzymogenes* [29]. Using its reported FT3SS counterparts as queries, we performed a local BLASTP search in the genome of strain OH11 and identified the respective counterparts (Figure 1A). Interestingly, the predicted FT3SS-like genes did not form an operon in the genome of strain OH11 (Figure 1A). To characterize whether these FT3SS-like genes have similar or distinct functions with their flagellar counterparts in regulating flagellar motility, we carried out a cross-complementation assay, in which each FT3SS-like gene from strain OH11 was cloned and introduced into the *Salmonella* mutants lacking the respective flagellar counterpart genes. We found that the FliR homolog restored the flagellum-mediated swimming motility of the *Salmonella fliR* null mutant to some extent after prolonged incubation at 30 °C, whereas the other homolog did not (Figure 1B; Figure S1), indicating that the FliR homolog is partially functional in *Salmonella* cells. This suggests that the FliR homolog forms a PMF-driven transmembrane export gate complex along with the *Salmonella* FlhA, FlhB, FliP and FliQ proteins.

To clarify whether FT3SS is functional in the non-flagellated strain OH11, we introduced the heterologous *fliC* gene, which encodes the flagellar filament protein named flagellin, into the non-flagellated strain OH11 to see whether the FT3SS-like genes could play a role in mediating flagellin export because this is the well-known function played by the canonical flagellar FT3SS genes [23]. For this purpose, we selected the FliC protein (PXO_06154, referred to here as FliC_Xoo_) from *X. oryzae* to generate a recombinant *fliC*_xoo_ product fused with a C-terminal Flag tag, which was cloned into a vector to drive its expression under a constitutive promoter. Using Western blotting with a specific anti-Flag antibody, we indeed found that the FliC_Xoo_-Flag fusion protein could be secreted into the culture supernatant of the wild-type OH11, while its secretion was completely abolished in either *flhB* or *fliI* knock-out strain. Cellular presence of FliC_xoo_-Flag was detected at similar levels in all tested strains withthe RNA polymerase ß-subunit serving as a control (Figure 1C). These results suggest that the non-flagellated *L. enzymogenes* OH11 produces a functional FT3SS that facilitates the export of the test heterologous FliC_Xoo_ protein. It is noteworthy that besides the FliR homolog, other predicted FT3SS-like proteins, FlhA, FlhB, FliP, FliQ and FliI, which failed to exert the flagellar protein export function in *Salmonella* (Figure S1), might have undergone functional divergence to match the native trait of strain OH11 in missing flagellum.

### 3.2. Several FT3SS-Like Genes in the Non-Flagellated L. enzymogenes OH11 Play Novel Functions to Affect Twitching Motility

**Figure 2:**
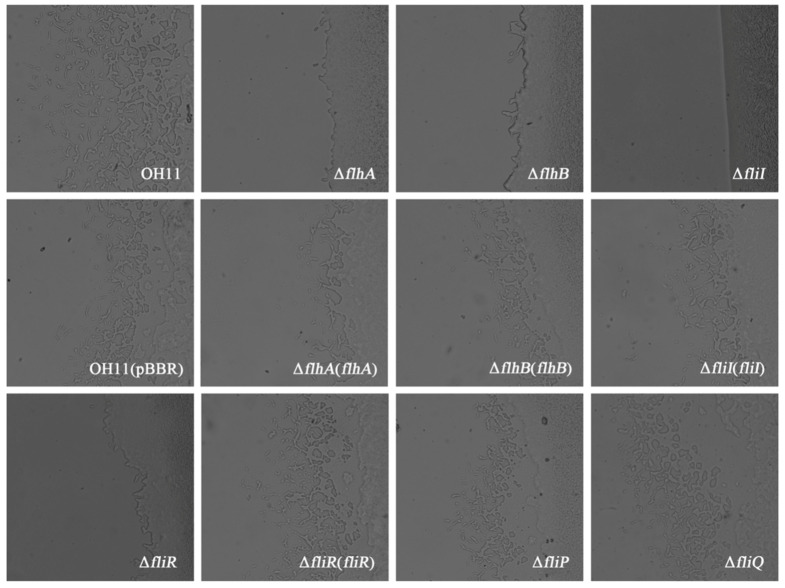
Involvement of the *FT3SS* genes in twitching motility in *L. enzymognes* OH11. In 1/10 tryptic soy broth (TSB) agar medium, wild-type OH11 exhibit twitching motility, as evidenced by the appearance of mobile cells at the colony margin, while the mutant strains, including Δ*flhA*, Δ*fllhB*, Δ*fliI*, and Δ*fliR* reveal no such capability. Complementing plasmid-borne *flhA*, *flhB*, *fliI*, or *fliR* gene back to each respective mutant rescued twitching motility. However, the Δ*fliP* and Δ*fliQ* mutants still show wild-type twitching motility.

The T4P pili facilitate twitching motility of *L. enzymogenes* OH11 on solid surfaces. The wild-type OH11 does not produce functional flagella because of the lack of flagellar genes (i.e., the flagellin gene). Thus, the motility of this strain (OH11) is not driven by the flagella. However, we found that the FT3SS is functional in the OH11 strain, raising the possibility that the FT3SS might contribute to the other motility systems (twitching motility) of strain OH11. Therefore, we next tested whether, upon the loss of flagellar motility, those remaining FT3SS-like genes in the non-flagellated strain OH11 would have an impact on twitching behavior. To test this, we individually generated in-frame deletion mutants of the FT3SS genes in the wild-type OH11 background and tested whether each gene disruption affectedtwitching motility on solid surfaces. As shown in Figure 2, the individual deletion mutation of *flhA*, *flhB*, *fliI*, and *fliR* in wild-type OH11 inhibited twitching motility, since no mobile cells at the colony margin of each mutant could be observed. Introduction of plasmid-borne *flhA*, *flhB*, *fliI*, or *fliR* back to each respective mutant rescued twitching motility. In contrast, the *fliP* and *fliQ* deletion mutants still exhibited wild-type twitching motility. These results suggest that the FlhA, FlhB, FliI and FliR homologs in the non-flagellated strain OH11 may have obtained a novel function to control twitching motility during the evolutionary process, whereas FliP and FliQ did not.

### 3.3. The FliI Homolog Affects PilA Secretion in L. enzymogenes OH11

**Figure 3:**
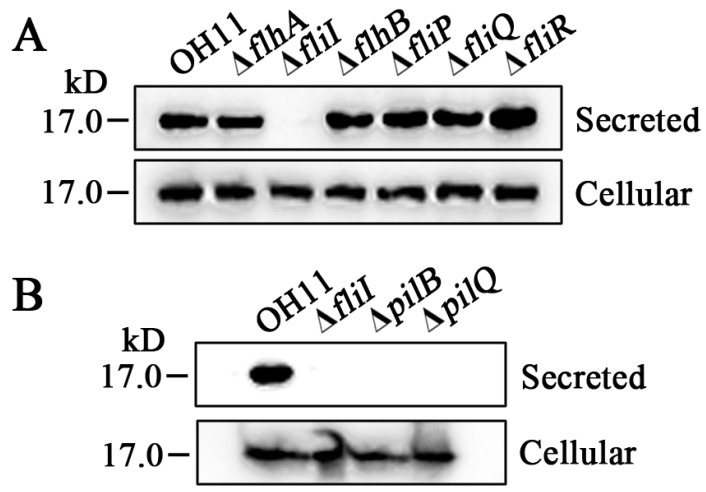
Differential contribution of FT3SS components to the secretion of PilA, the major pilin of type IV pilus in the non-flagellated *L. enzymognes* OH11. (**A**) Western blotting showing inactivation of FliIfully blocked PilA secretion. The coding gene of PilA was fused with a Flag-tag and introduced into wild-type OH11 and the FT3SS-defective mutants (Δ*flhA*, Δ*fllhB*, Δ*fliI*, Δ*fliP*, Δ*fliQ*, or Δ*fliR*). Strains were cultivated in 1/10 TSB, and cells were collected at OD_600_, 1.5. Except for the *fliI* mutant (Δ*fliI*), the signal of PilA-Flag determined by anti-Flag antibody was detected both in the culture supernatant of wild-type OH11 and the remaining five mutants, while cellular presence of PilA-Flag was observed in all test strains. (**B**) Western blot verification of PilA secretion was dependent both on the FliI and on two other pilus apparatus proteins, PilB (a pilus ATPase that provides energy via ATP hydrolysis to promote pilus extension) and PilQ (which forms an outer membrane secretin pore comprising multiple monomers to facilitate pilus extraction).

What is the mechanism by which the FT3SS genes modulate twitching motility in the non-flagellated strain OH11? In our earlier study [7], we showed that the cellular expression and the secretion of PilA, the major pilin, isessential for the formation of twitching motility in strain OH11, and we thus investigated whether the FT3SS components were utilized in such a manner to affect twitching motility. For this purpose, a plasmid containing the PilA-Flag fusion was introduced into wild-type OH11 and its deletion mutant derivatives. The transformed strains were cultivated in 1/10 TSB with cells collected at OD_600_ 1.5, followed by Western blotting via anti-Flag antibody. The secretion of the PilA-Flag fusion into the culture medium was not detected in the *fliI* mutant, although its intracellular amount was similar tothat of wild type. Under the same test conditions, the PilA-Flag fusion was detected in the culture supernatants of the *flhA*, *flhB* and *fliR* mutants in a way similar to thewild-type strain OH11 (Figure 3A). As controls, the presence of the PilA-Flag fusion was also detected in the culture supernatant and cellular fractions of the *fliQ* and *fliP* mutants (Figure 3A), both of which produced the wild-type twitching motility (Figure 2). These results imply that while FlhA, FlhB, FliI, and FliR all participate in the formation of twitching motility, only FliI has a remarkable impact on the secretion of PilA-Flag.

To validate the above findings, we further chose two *L. enzymogenes* T4P-defective mutants, Δ*pilB* and Δ*pilQ*, as additional strains, since both fail to produce surface-attached T4P according to our earlier study [7]. As expected, we could only detect the intracellular presence of PilA-Flag in both mutants, but no signal was observed in their culture supernatants (Figure 3B), which agreed with those of the *fliI*mutant (Figure 3B). These results lead to a plausible hypothesis that the flagellar FliI ATPase in the non-flagellated strain OH11 has likely evolved to act as PilB-like ATPase to promote PilA secretion, there by contributing totwitching motility in *L. enzymogenes*.

## 4. Discussion

In bacterial physiology, the bacterial flagellum is one of the best-studied surface-attached appendages, whose assembly relies on a wide array of flagellar proteins [10,22]. The translocation of flagellar proteins from cytoplasm across the cytoplasmic membrane is governed by FT3SS [14], which is generally recognized as the main factor in controlling flagellar assembly and hence affects flagellum-driven motility in flagellated bacteria [14,15,16,17,18,19,20,21,22]. Here, we demonstrated an intriguing case showing that the FT3SS-like geneswere encodedon the genome of a non-flagellated environmentally ubiquitous bacterium and acquired novel functions to enhance twitching motility. Such findings expand our current knowledge on the functional evolution or divergence of the conserved FT3SS genes from flagellated to non-flagellated bacteria, which may provoke the interests of scientists studying various bacterial species with and without flagella.

However, one may question why the FT3SS-like genesare evolutionarily retained in the non-flagellated *L. enzymogenes*. In this bacterium, our experimental data indicate that the retained FT3SS-like gene products possibly assemble into a functional FT3SS system that is able to mediate the export of the heterologous flagellin, as with the case played by the canonical *FT3SS* genes in flagellated bacteria [12]. This finding raises a possibility that the FT3SS-like genes in the non-flagellated *L. enzymogenes* might have a coordinated role in mediating export of native, yet unidentified flagellar or non-flagellar proteins. We are currently testing this hypothesis. The findings that several FT3SS-like genes in strain OH11 playing a role in boosting twitching motility is interesting and ecologically relevant, because such existing twitching behavior may help the non-flagellated *L. enzymogenes* move and prey upon fungi for nutrients.

One may further ask how the FT3SS-lilke genes diverge to affect twitching motility in the non-flagellated *L. enzymogenes*. As documented previously [7], twitching motility is controlled by the T4P, which is assembled by the major pilin, PilA, and other minor pilins. PilB is an ATPase to hydrolyze ATP to drive pilus assembly, forming a pilus that extends to the cell surface via an outer membrane secretin pore comprised of multiple PilQ monomers [29,30,31]. The non-flagellated *L. enzymogenes* also utilizes this similar strategy to perform twitching motility, because it has been shown that the inactivation of either PilB or PilQ abolishes the secretion of PilA, thereby inhibiting twitching motilityof strain OH11 [7].Here, we showed that among the four FT3SS components (FlhA, FlhB, FliI, and FliR), FliI is the only component that is directly involved in PilA secretion. In flagellated bacteria, FliI is the Walker-type ATPase of FT3SS which hydrolyzes ATP to allow the transmembrane export gate complex made up of FlhA, FlhB, FliP, FliQ and FliR to drive the export of flagellar proteins from the cytoplasm to the distal end of growing flagellar structure in a PMF-dependent manner [14,16,19]. The crystal structure of the *Salmonella* FliI shows extensive structural similarities to α and β subunits of F1-ATPase [32,33]. The FliI ATPase forms a homo-hexamer to hydrolyze ATP at an interface between FliI subunits in a way similar to F1-ATPase [34]. PilB and PilQ also form a hexameric ring-like structure as seen in F1-ATPase and FliI [35]. Moreover, bioinformatic analyses via the online software [36] led to the observation that the *L. enzymogenes* FliI homolog belonged to the F1-ATPase family; it possessed a nucleotide-binding domain and a beta-barrel domain, ranging from the sequence positions of 162 to 372 and 40 to 106, respectively. This information, along with our present findings, suggests that other than the canonical PilB, FliI in the non-flagellated *L. enzymogenes* has evolved to act as the second ATPase hexamer to contribute to twitching motility of *L. enzymogenes* via affecting PilA secretion. In this regard, the FliI–PilA pair is postulated to act as a bridge to indirectly link FT3SS with pilus assembly in the non-flagellated *L. enzymogenes.* This finding is also in agreement with a recent study showing that different flagellar gene mutations affect levels of pilus production in *Caulobacter crescentus*, another flagellated bacterium [37]. However, under the current test conditions, this mode of action was possibly not applied by the remaining three FT3SS components (FlhA, FlhB, and FliR) in *L. enzymogenes*, because although they were required for twitching motility of the strain OH11, they did not in fact affect PilA secretion. This datum reveals the possibility that an additionally uncharacterized mechanism might exist for FT3SS components to control the twitching motility in *L. enzymogenes*.

Overall, we provided some intriguing evidence showing how some FT3SS-like genes have adopted altered function during the evolutional process to confer adaptive advantage for a non-flagellated bacterium. This study thus increases our understanding ofthe functional divergence of flagellar genes from flagellated to non-flagellated bacteria.

## 5. Conclusions

In the present study, we report that several FT3SS-like genes—which are potentially vestigial in the non-flagellated biocontrol bacterium, *L. enzymogenes* OH11—appear to be required for type-IV-pilus-driven twitching motility. We furthermore suggest that, upon the loss of flagella-driven motility, several of the remaining FT3SS-like genes have acquired a novel function to control twitching motility. The flagellar FliI ATPase in particular would have evolved to act as a PilB-like ATPase involved in PilA secretion. These findings reveal that the FT3SS genes have undergone functional divergence between flagellated and non-flagellated bacteria, supporting the recent notion that FT3SS plays roles in bacterial physiology beyond flagella production.

## Supplementary Materials

The following are available online at https://www.mdpi.com/2218-273X/10/5/733/s1, Figure S1: Cross-complementation of the *L. enzymogenes* FT3SS-like genes in the *Salmonella* mutants lacking the respective flagellar counterparts, Table S1: Strains and plasmids used in this study title, Table S2: Primers used in this study.
