# Supplementary material for: The Homologous Components of Flagellar Type III Protein Apparatus Have Acquired a Novel Function to Control Twitching Motility in a Non-Flagellated Biocontrol Bacterium
Source: Biomolecules. 2020 May 7;10(5):733. doi: 10.3390/biom10050733 (PMC7277350; doi:10.3390/biom10050733)
Supplement: Supplementary file 1 [file biomolecules-10-00733-s001.pdf]

Supplementary material

The homologous components of flagellar type III protein apparatus have acquired a novel function to control twitching motility in a non-flagellated biocontrol bacterium

Alex M. Fulano[1], Danyu Shen[1], Miki Kinoshita[2], Shan-Ho Chou[3], Guoliang Qian[1*]

Table S1. Strains and plasmids used in this study

Strains and plasmids	Characteristics[a]	source
Lysobacter enzymogenes		
OH11	Wild type (CGMCC No. 1978), Km^R	[1]
Δ*flhA*	*flhA* in-frame deletion mutant, Km^R	This study
Δ*flhB*	*flhB* in-frame deletion mutant, Km^R	This study
Δ*fliI*	*fliI* in-frame deletion mutant, Km^R	This study
Δ*fliP*	*fliP* in-frame deletion mutant, Km^R	This study
Δ*fliQ*	*fliQ* in-frame deletion mutant, Km^R	This study
Δ*fliR*	*fliR* in-frame deletion mutant, Km^R	This study
Δ*pilA*	*pilA* in-frame deletion mutant, Km^R	[2]
Δ*pilB*	*pilB* in-frame deletion mutant, Km^R	[2]
OH11(pBBR)	Wild type harboring plasmid pBBR1-MCS5, Gm^R, Km^R	This study
OH11(*pilA*)	Wild type harboring plasmid pBBR-*pilA*, Gm^R, Km^R	This study
Δ*flhA*(*flhA*)	Δ*flhA* harboring pBBR-*flhA*, Gm^R, Km^R	This study
Δ*flhB*(*flhB*)	Δ*flhB* harboring pBBR-*flhB*, Gm^R, Km^R	This study
Δ*fliI*(*fliI*)	Δ*fliI* harboring pBBR-*fliI*, Gm^R, Km^R	This study
Δ*fliR*(*fliR*)	Δ*fliR* harboring pBBR-*fliR*, Gm^R, Km^R	This study
Δ*flhA*(*pilA*)	Δ*flhA* harboring pBBR- *pilA*, Gm^R, Km^R	This study
Δ*flhB*(*pilA*)	Δ*flhB* harboring pBBR- *pilA*, Gm^R, Km^R	This study
Δ*fliI*(*pilA*)	Δ*fliI* harboring pBBR- *pilA*, Gm^R, Km^R	This study
Δ*fliR*(*pilA*)	Δ*fliR* harboring pBBR- *pilA*, Gm^R, Km^R	This study
Δ*pilB*(*pilA*)	Δ*pilB* harboring pBBR- *pilA*, Gm^R, Km^R	This study
Δ*pilQ*(*pilA*)	Δ*pilQ* harboring pBBR- *pilA*, Gm^R, Km^R	This study
Escherichia coli DH5α	Host strain for molecular cloning	Laboratory collection
Xanthomonas oryzae PX099A	Source of the canonical $FliC_{xoo}$	Laboratory collection
Salmonella		
Δ*flhA*$_{sal}$	*flhA* in-frame deletion mutant	[3]
Δ*flhB*$_{sal}$	*flhB* in-frame deletion mutant	[3]
Δ*fliI*$_{sal}$	*fliI* in-frame deletion mutant	[3]
Δ*fliP*$_{sal}$	*fliP* in-frame deletion mutant	[3]
Δ*fliQ*$_{sal}$	*fliQ* in-frame deletion mutant	[3]
Δ*fliR*$_{sal}$	*fliR* in-frame deletion mutant	[3]

Δ*flhA*$_{sal}$ (pTrc99A-FlhA)	Δ*flhA*$_{sal}$ harboring pBBR-*flhA*, Amp^R	This study
Δ*flhB*$_{sal}$ (pTrc99A-FlhB)	Δ*flhB*$_{sal}$harboring pBBR-*flhB*, Amp^R	This study
Δ*fliI*$_{sal}$ (pTrc99A-FliI)	Δ*fliI*$_{sal}$ harboring pBBR-*fliI*, Amp^R	This study
Δ*fliP*$_{sal}$ (pTrc99A-FliP)	Δ*fliPA*$_{sal}$ harboring pBBR-*fliR*, Amp^R	This study
Δ*fliQ*$_{sal}$ (pTrc99A-FliQ)	Δ*fliQ*$_{sal}$ harboring pBBR-*fliQ*, Amp^R	This study
Δ*fliR*$_{sal}$ (pTrc99A-FliR)	Δ*fliR*$_{sal}$ harboring pBBR-*fliR*, Amp^R	This study
**Plasmids**		
pEX18GM	Suicide vector with a *sacB* gene, Gm^R	[4]
pBBR1-MCS5	Broad-host-range vector with a Plac promoter, Gm^R	[5]
pEX18-*flhA*	pEX18GM with two flanking fragments of *flhA*, Gm^R	This study
pTrc99A-FlhA	An expression vector in *Salmonella*, Amp^R	[3]
pEX18-*flhB*	pEX18GM with two flanking fragments of *flhB*, Gm^R	This study
pEX18-*fliI*	pEX18GM with two flanking fragments of *fliI*, Gm^R	This study
pEX18-*fliR*	pEX18GM with two flanking fragments of *fliR*, Gm^R	This study
pEX18-*fliP*	pEX18GM with two flanking fragments of *fliP*, Gm^R	This study
pEX18-*fliQ*	pEX18GM with two flanking fragments of *fliQ*, Gm^R	This study
pBBR-*flhA*	pBBR1-MCS5 cloned with a 2385-bp fragment containing intact *flhA* and its predicted promoter, Gm^R	This study
pBBR-*flhB*	pBBR1-MCS5 cloned with a 1557-bp fragment containing intact *flhB* and its predicted promoter, Gm^R	This study
pBBR-*fliI*	pBBR1-MCS5 cloned with a 1562-bp fragment containing intact *fliI*, FLAG tag and its predicted promoter, Gm^R	This study
pBBR-*fliR*	pBBR1-MCS5 cloned with a 1277-bp fragment containing intact *fliR* and its predicted promoter, Gm^R	This study
pBBR- *pilA*	pBBR1-MCS5 cloned with a 737-bp fragment containing a PilA-Flag tag, Gm^R	This study
pTrc99A-FlhA	pTrc99A cloned with the coding region of *flhA* of strain OH11, Amp^R	This study
pTrc99A-FlhB	pTrc99A cloned with the coding region of *flhB* of strain OH11, Amp^R	This study
pTrc99A-FliI	pTrc99A cloned with the coding region of *fliI* of strain OH11, Amp^R	This study
pTrc99A-FliP	pTrc99A cloned with the coding region of *fliP* of strain OH11, Amp^R	This study
pTrc99A-FliQ	pTrc99A cloned with the coding region of *fliQ* of strain OH11, Amp^R	This study

[a]Km^R, Gm^R and Amp^R: kanamycin-, gentamicin, and Ampicillin-resistance, respectively

Table S2. Primers used in this study

Primer	Sequence[a] (5′-3′)	Purpose
**In-frame deletion**		
flhA-F1	CGGAATTCGATCGCCAAGAGCCAGGAAC (*Eco*RI)	To amplify a 794-bp upstream homologue arm of *flhA*
flhA-R1	GGGTTTGAAGGAGACGGGCGCGACGATGGTGGGGGCAAGC	
flhA-F2	GCTTGCCCCCACCATCGTCGCGCCCGTCTCCTTCAAACCC	To amplify a 799-bp downstream homologue arm of *flhA*
flhA-R2	GCTCTAGACTGTGCGGATTCGATGCGGCCGACGATGGTGGG GGCAAGC (*Xba*I)	
flhB-F1	CCGGAATTCCGTTCCTGGTGTTCCTCGAC (*Eco*RI)	To amplify a 538-bp upstream homologue arm of *flhB*
flhB-R1	CGCCGACCCAGAAAAAGATCGGTCCTGCATTGGGCCGAAG	
flhB-F2	CTTCGGCCCAATGCAGGACCGATCTTTTTCTGGGTCGGCG	To amplify a 464-bp downstream homologue arm of *flhB*
flhB-R2	GCTCTAGACCAGGCATCGGCGTTGAGGG (*Xba*I)	
flip-F1	CGGAATTCGGCTCACGGTCAAGGAACTG (*Eco*RI)	To amplify a 526-bp upstream homologue arm of *fliP*
flip-R1	GCGGCGAGGTCATTCATTTGCGATGGCGGACGAAGAAGGC	
flip-F2	GCCTTCTTCGTCCGCCATCGCAAATGAATGACCTCGCCGC	To amplify a 645-bp downstream homologue arm of *fliP*
flip-R2	GCTCTAGAGGCAGCGAATGGAAATAGTG (*Xba*I)	
fliQ-F1	CGGAATTCGAACATCCTGCTCTCGCTGG (*Eco*RI)	To amplify a 336-bp upstream homologue arm of *fliQ*
fliQ-R1	CCCAAGCTTGCCTGTTTGGTCATTTCGAG (*Hin*dIII)	
fliQ-F2	CCCAAGCTTCGCCGACCGCATCTTCCTCG (*Hin*dIII)	To amplify a 363-bp downstream homologue arm of *fliQ*
fliQ-R2	GCTCTAGACCCAGAACACCATGCCGAAC (*Xba*I)	
fliR-F1	CGGAATTCCACGAGGACGACACCGCGAC(*Eco*RI)	To amplify a 631-bp upstream homologue arm of *fliR*
fliR-R1	CCCAAGCTTCGGCGAGGCGATCAGGAAAC(*Hin*dIII)	
fliR-F2	CCCAAGCTTCCTTGGTGATGCTGACGCTC(*Hin*dIII)	To amplify a 923-bp downstream homologue arm of *fliR*
fliR-R2	GCTCTAGACGTAATGGGTGGGGTTGGTG(*Xba*I)	
fliI-F1	CCGGAATTCTTCTCCAGCGCCTCCAGCCT (*Eco*RI)	To amplify a 389-bp upstream homologue arm of *fliI*
fliI-R1	CCCAAGCTTCGGATGAACAAGCAACGCT (*Hin*dIII)	
fliI-F2	CCCAAGCTTCGACCACTTCGGCGGCGAG (*Hin*dIII)	To amplify a 457-bp downstream homologue arm of *fliI*
fliI-R2	CGGGGTACCCTGCGTGGTGGTGTCGGAAG (*Kpn*I)	
**Complementation and heterologous expression**		
flhA-F	CCCAAGCTTCACCAACCCCACCCATTACG (*Hin*dIII)	To amplify 2409-bp fragment containing the intact *flhA*
flhA-R	CGGAATTCTCACTTATCGTCGTCATCCTTGTAATCGGCGGCG ACGGGTTTGA (*Eco*RI)	
flhB-F	CCCAAGCTTGATGGACGCAATGTAGCCCC (*Hin*dIII)	To amplify 1331-bp fragment containing the intact *flhB*
flhB-R	CGGAATTCTCACTTATCGTCGTCATCCTTGTAATCGCGCGGC TCGTCGTCGA (*Eco*RI)	
fliI-F	CCCAAGCTT CTGCGTGGTGGTGTCGGAAG (*Hin*dIII)	To amplify 1562-bp fragment containing the intact *fliI*
fliI-R	CGGAATTCTCACTTATCGTCGTCATCCTTGTAATCTCCGAAC AACCTGCGCA (*Eco*RI)	
flip-F	CCCAAGCTTGGCTCACGGTCAAGGAACTG (*Hin*dIII)	To amplify 1101-bp fragment containing the intact *fliP*
flip-R	CGGAATTCTCACTTATCGTCGTCATCCTTGTAATCTTTGAAGC TGAGCACCA (*Eco*RI)	
fliQ-F	CCCAAGCTTCACCGTCAGCGAACTCACCG (*Hin*dIII)	To amplify 676-bp fragment containing the intact *fliQ*
fliQ-R	CGGAATTCTCACTTATCGTCGTCATCCTTGTAATCGTGCCGG ATCATGCCGG (*Eco*RI)	
fliR-F	CCCAAGCTTGAAGGACACGACGAGCAGGC (*Hin*dIII)	To amplify 1300-bp fragment containing the intact *fliR*
fliR-R	CGGAATTCTCACTTATCGTCGTCATCCTTGTAATCTGGCGCG CCCGCCGGGC (*Eco*RI)	
pilA-F	CCCAAGCTTCATCGCAAATCAACCCCCAC(*Hin*dIII)	To amplify 737-bp fragment containing the *pilA*-Flag fusion

pilA-R	CGGAATTCTTACTTATCGTCGTCATCCTTGTAATCGGTCGCA GCCTTGGTGC(*Eco*RI)	
fliC$_{Xoo}$ F2	CCCAAGCTTATGTCGCTGAACGCTCAGCG (*Hin*dIII)	To amplify *fliC*$_{Xoo}$ terminally fused with FLAG tag
fliC$_{Xoo}$ F2	CGGAATTCTTACTTATCGTCGTCATCCTTGTAATCCTGCAGCA GGCTCAGCA(*Eco*RI)	

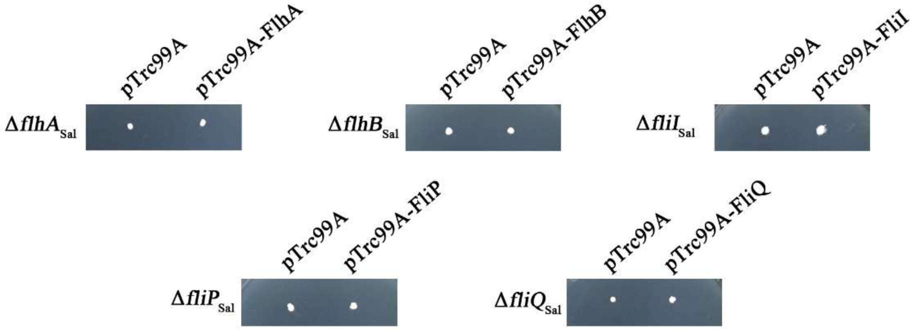

Figure S1. Cross-complementation of the *L. enzymogenes* FT3SS-like genes in the *Salmonella* mutants lacking the respective flagellar counterparts. The FlhA, FlhB, FliP, FliQ and FliI from strain OH11 failed to restore the swimming motility of the respective *Salmonella* mutants, indicated by Δ*flhA*$_{sal}$, Δ*flhB*$_{sal}$, Δ*fliP*$_{sal}$, Δ*fliQ*$_{sal}$ and Δ*fliI*$_{sal}$, respectively. The photograph was taken after incubation of the transformed strain at 30ºC for 24 hours.

References

1. Qian, G.L.; Hu. B.S.; Jiang, Y.H.; Liu, F.Q. Identification and characterization of *Lysobacter enzymogenes*as a biological control agent against some fungal pathogens. *Agr Sci China.* **2009**, *8*, 68-75.
2. Xia, J.; Chen, J.J.; Chen, Y.; Qian, G.L.; Liu, F.Q. Type IV pilus biogenesis genes and their roles in biofilm formation in the biological control agent *Lysobacter enzymogenes* OH11. *Appl Microbiol Biotechnol*. **2018**, *102*, 833-846.
3. Minamino, T.; Namba, K. Distinct roles of the FliI ATPase and proton motive force in bacterial flagellar protein export. *Nature.* **2008**, *451*, 485-488.
4. Hoang, T.T.; Karkhoff-Schweizer, R.R.; Kutchma, A.J; Schweizer, H.P. A broad-host-range Flp-FRT recombination system for site-specific excision of chromosomally-located DNA sequences: application for isolation of unmarked *Pseudomonas aeruginosa* mutants. *Gene*. **1998**, *212*, 77-86.
5. Kovach, M.E.; Elzer, P.H; Hill, D.S; Robertson, G.T.; Farris, M.A.; Roop, R.M.; Peterson, K.M. Four new derivatives of the broad-host-range cloning vector pBBR1MCS, carrying different antibiotic-resistance cassettes. *Gene*. **1995**, *166*, 175-176.
